# Novel Multi-Parametric Sensor System for Comprehensive Multi-Wavelength Photoplethysmography Characterization

**DOI:** 10.3390/s23146628

**Published:** 2023-07-24

**Authors:** Joan Lambert Cause, Ángel Solé Morillo, Bruno da Silva, Juan C. García-Naranjo, Johan Stiens

**Affiliations:** 1 Department of Electronics and Informatics (ETRO), Vrije Universiteit Brussel (VUB), 1050 Brussels, Belgium; angelsm@etrovub.be (Á.S.M.); jstiens@etrovub.be (J.S.); 2 Department of Biomedical Engineering, Universidad de Oriente, Santiago de Cuba 90500, Cuba; 3 Centre of Medical Biophysics, Universidad de Oriente, Santiago de Cuba 90500, Cuba; jcgnaranjo@uo.edu.cu

**Keywords:** photoplethysmography, MW-PPG, multi-wavelength, contact force, temperature, PSoC

## Abstract

Photoplethysmography (PPG) is widely used to assess cardiovascular health. However, its usage and standardization are limited by the impact of variable contact force and temperature, which influence the accuracy and reliability of the measurements. Although some studies have evaluated the impact of these phenomena on signal amplitude, there is still a lack of knowledge about how these perturbations can distort the signal morphology, especially for multi-wavelength PPG (MW-PPG) measurements. This work presents a modular multi-parametric sensor system that integrates continuous and real-time acquisition of MW-PPG, contact force, and temperature signals. The implemented design solution allows for a comprehensive characterization of the effects of the variations in these phenomena on the contour of the MW-PPG signal. Furthermore, a dynamic DC cancellation circuitry was implemented to improve measurement resolution and obtain high-quality raw multi-parametric data. The accuracy of the MW-PPG signal acquisition was assessed using a synthesized reference PPG optical signal. The performance of the contact force and temperature sensors was evaluated as well. To determine the overall quality of the multi-parametric measurement, an in vivo measurement on the index finger of a volunteer was performed. The results indicate a high precision and accuracy in the measurements, wherein the capacity of the system to obtain high-resolution and low-distortion MW-PPG signals is highlighted. These findings will contribute to developing new signal-processing approaches, advancing the accuracy and robustness of PPG-based systems, and bridging existing gaps in the literature.

## 1. Introduction

Photoplethysmography (PPG) is a non-invasive and cost-effective optical technique that measures changes in blood volume. It has been successfully proven in various applications, such as measuring heart and respiratory rates, evaluating arterial and venous diseases, and estimating arterial oxygen saturation (SpO${}_{2}$) [1]. The PPG signal consists of AC and DC components. The AC component reflects changes in blood volume synchronized with cardiac cycles. In contrast, the DC component represents the baseline blood volume level, affected by non-pulsatile tissue absorption and low-frequency variations associated with respiration, thermoregulation, and sympathetic activity [2,3]. PPG measurements require a light source (emitter) and a photo-detector (receiver) to measure blood volume changes at specific wavelengths. The PPG waveform is generated through the interaction of light with various tissue layers, wherein optical phenomena such as scattering, absorption, and reflection occur [4]. The size and curvature of the banana-shaped scattering pattern of the photons along their propagation path are wavelength-dependent. Hence, the distance between the light source and the photo-detector determines the propagation depth inside the tissue from which photons are collected [5].

PPG measurements can be performed in two modes, namely transmission and reflection. Still, the reflection mode is more flexible since more wavelengths can be used and due to its applicability to any part of the body, which makes it more suitable for non-invasive long-term monitoring wearable devices [6]. PPG measurements at multiple wavelengths (MW-PPG) can provide a more comprehensive view of hemodynamic changes in the body, such as changes in arterial SpO${}_{2}$, pulse transit time (PTT), and peripheral perfusion. This detailed information is crucial for developing high-precision and reliable non-invasive medical monitoring applications, as emphasized in [7].

The information extracted from the PPG signal depends on signal features and their interrelation. The PPG signal includes essential features based on the original PPG waveform, combined features, and derivative features [8]. However, the estimation of these attributes is influenced by several factors, which can be classified into three categories: cardiovascular, biological, and acquisition [9,10,11]. Acquisition factors are related to the properties and intensity of the emitted light [12], ambient light [13], photo-detector sensitivity [14], measurement point [15], temperature [16], motion artifacts [17], and contact force (CF) between the sensor and the skin [18], among others. Temperature fluctuations can cause changes in blood flow and alterations in the PPG waveform [16,19,20,21]. At the same time, inadequate or excessive CF can impact the quality of the PPG signal, leading to motion artifacts or tissue compression [18,22,23,24,25]. These factors can influence the accuracy and reliability of PPG measurements, making it challenging to extract useful physiological information.

Recent studies have highlighted the significance of considering factors such as CF and temperature in PPG signal analysis for accurate and reliable measurements. Chandrasekhar et al. [25] emphasize the importance of CF in PPG-based blood pressure (BP) control, noting that changes in CF can lead to errors in BP estimations, mainly when using the fingertip for Pulse Arrival Time (PAT) detection. Additionally, Maeda et al. [26] compared green and infrared PPG signals and ECG during heat stress to investigate the influence of temperature on PPG signals, emphasizing the reliability of green PPG signals for pulse rate measurements, albeit with potential dependence on other factors. Meanwhile, Budidha and Kyriacou [27] explored the reliability of ear canal PPG sensors in cold temperatures, highlighting the limitations of peripheral perfusion and the impact of the thermal state of the subjects on pulse oximeters.

The use of MW-PPG has broadened the field of application of traditional PPG and, in many cases, improved it [28,29]. Shorter wavelengths, such as green light, are preferred for monitoring heart rate during daily activities due to lower susceptibility to artifacts [30], low temperatures [26], and inadequate probe contact [25]. MW-PPG methods have improved the accuracy of blood pressure measurement [31,32] by calculating PTT derived from different wavelengths. MW-PPG has also promoted research in other fields of interest in the analysis of blood components, such as the non-invasive detection of glucose levels in blood [33] and drug delivery control [34]. While MW-PPG has shown potential to improve the accuracy and reliability of PPG measurements, further research is needed to optimize their use [7].

Several review studies discussing the main influencing factors in PPG have been published [35,36,37]. The great range of these influencing factors hinders the ability to measure and control all of them simultaneously, introducing a big variability in how PPG-based studies are conducted. Since these influencing factors are not properly accounted for with a standardized protocol, inhomogeneous results with poor reproducibility are common [36,37]. Any strategy to address this issue should consider the complex interrelation between PPG features and external factors that affect PPG measurements. Therefore, a thorough characterization of the relevant factors influencing the intended application is required to enable standardization and minimize variability among reference studies and devices. To achieve reliable and robust PPG-based monitoring systems, it is crucial to consider the complex interrelation between PPG features and external factors affecting PPG measurements. A design process focused on the relevant factors influencing the intended application will lead to standardization, minimizing the variability among referent studies and devices.

This work presents the development of a multi-parametric sensor system to comprehensively characterize the effects of CF and temperature on MW-PPG measurements. The design, based on a Programmable System on Chip (PSoC) processor, consists of functional blocks including the Optical Front-End (OFE), Analog Front-End (AFE), Auxiliary Sensor (AUX), and Digital Signal Processing (DSP). The OFE evaluates PPG signals using five wavelengths (470, 525, 590, 631, and 940 nm), while the AUX block incorporates a CF sensor and a temperature sensor. The primary objective of the system is to provide complete and high-quality raw data to assess the evolution of the PPG signal contour at different vascular layers and serve as an additional tool in developing and testing new signal processing approaches.

Signal processing through a PSoC enables the evaluation of different AFE and DSP configurations without significant hardware board changes, increasing flexibility. The system is modular and easy to integrate with different measurement platforms. It allows for modifications in the number of wavelengths and the spatial arrangement of the optical elements of the OFE, as well as the incorporation of other sensors to evaluate additional factors. A Dynamic DC Cancellation (DDCC) circuit removes DC for each wavelength separately and can be adjusted to meet experimental needs. A preliminary evaluation showed that the system accurately captures PPG signals without introducing distortion or altering the original morphology. The system also demonstrated the ability to perform multi-parametric measurements, including temperature measurements, CF detection, and MW-PPG.

The primary contributions of this work are as follows:

- Comprehensive characterization of CF and temperature effects on MW-PPG measurements, especially evaluating the evolution of the multi-wavelength signal contour.
- System modularity, which allows for the inclusion of additional sensors and the testing of new hardware configurations.
- A novel tool for designing innovative signal processing techniques and algorithms to improve the interpretation of the MW-PPG signal.

The architecture and implementation are, respectively, described in Section 2, while the measurements made to evaluate the design are presented and discussed in Section 3. Future work is presented in Section 4, and the conclusions are drawn in Section 5.

## 2. Design and Configuration of the Multi-Parametric Sensor System

To understand how variations in CF and skin temperature affect the MW-PPG signal waveform, the following design requirements for the initial version of the multi-parametric sensor system have been identified:

- The system must be capable of acquiring, conditioning, and delivering accurate synchronized raw data, including signals such as MW-PPG, skin temperature, and CF.
- The system should be programmable, modular, and expandable, allowing for the reconfiguration of hardware resources to suit specific measurement requirements.

The system must deliver accurate and synchronized multi-parametric data to improve our understanding of how temperature and CF variations affect the MW-PPG signal. By knowing the degree of distortion of the PPG signal induced by temperature and CF, more accurate techniques and algorithms can be developed to interpret the relevant physiological information extracted from the PPG signal more reliably, reducing the effect of these disturbances. The architecture and implementation of the proposed design will be described below.

### 2.1. Sensor System Architecture

**Figure 1:**
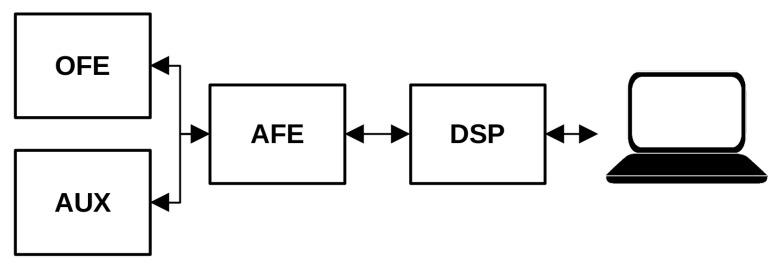
Functional block diagram of the proposed multi-parametric sensor system. The system comprises four key components: Optical Front-End (OFE), Analog Front-End (AFE), Auxiliary Sensors (AUX), and Digital Signal Processing (DSP).

The proposed system comprises four main functional blocks, depicted in Figure 1. Combining these four components, the multi-parametric sensor system offers an integrated solution for obtaining precise and comprehensive measurements. The Optical Front-End (OFE) module serves as the first component of the system. It captures optical signals and comprises light emitters and detectors to measure the MW-PPG signals. The system incorporates Auxiliary Sensors (AUX) to enhance the primary MW-PPG measurements to provide supplementary contextual information. This block integrates signals from these sensors to measure the pertinent physical variables impacting the behavior of the PPG waveform. While this work considers temperature and CF sensors, there is also room for incorporating additional auxiliary sensors. The Analog Front-End (AFE) plays a vital role in conditioning and amplifying the signals received from the OFE and AUX blocks. It ensures the signals are appropriately prepared for further processing, guaranteeing accurate and reliable data analysis. Furthermore, the Digital Signal Processing (DSP) block digitizes, pre-processes, formats, and transmits the signal to a computer. The OFE and AUX blocks are designed to be flexible to allow for different sensor configurations. The AFE and DSP are integrated into a CY8CKIT-059 development kit with a 32-bit ARM Cortex-M3 CPU, which reduces the need for external components and enables the efficient testing of measurement configurations.

**Figure 2:**
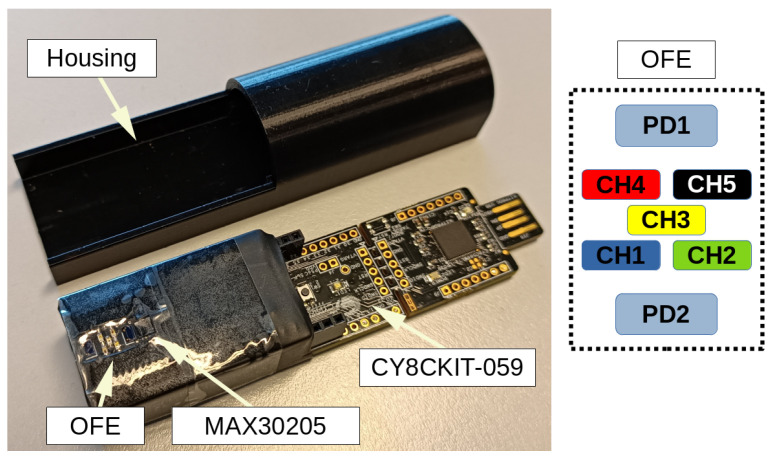
Experimental setup (**left**) and spatial distribution of OFE optical elements (**right**).

Figure 2 shows the experimental setup used for proof-of-concept and sensor testing across all body sites. The OFE design evaluated in this work includes five LED channels (CH1 to CH5) and two independent PDs (PD1 and PD2). The wavelengths assigned to CH1 through CH5 are 470, 525, 590, 631, and 940 nm, respectively. The LED arrangement is such that the shortest wavelength LED is placed closest to PD2, and the longest wavelength LED is located furthest from PD2. This configuration ensures that the 590 nm LED is equidistant from both PDs. Osram Opto Semiconductors SFH2703 high-speed, high-sensitivity PDs are used, with a spectral sensitivity range of 400–1100 nm. For visible and near-infrared (NIR) wavelengths, Vishay Semiconductors VLMx1300 series LEDs and Würth Elektronik eiSos GmbH & Co WL-SICW LEDs were selected, respectively. Control of the 5 LED channels is accomplished by a Texas Instruments TLC5940 chip that uses an internal 6-bit constant current source to regulate the bias current of each LED individually. The selected operating range is 0.00–35.50 mA, with a resolution of 0.55 mA, which can be extended through a bias current programming resistor. The AUX block includes a FlexiForce A101 force sensor unit from Tekscan Inc. and a MAX30205 medical-grade temperature sensor from Analog Devices. Figure 2 shows that the MAX30205 is placed on the same PCB as the OFE, located 1.5 mm from PD2. A transparent tape covers the OFE, while a 3D-printed casing protects the electronics.

**Figure 3:**
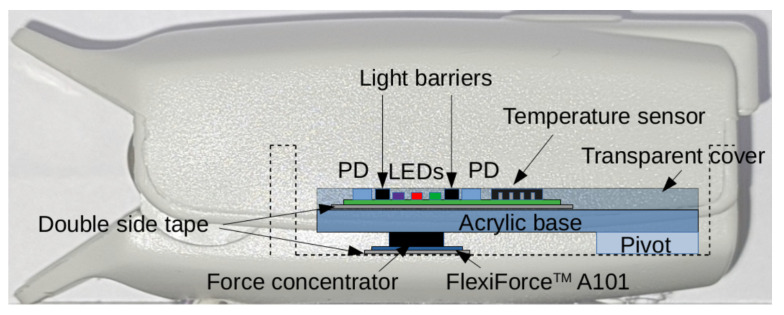
Internal details of the experimental setup for measuring multi-parametric signals in vivo at the fingertips. The plastic casing that contains the PSoC and the rest of the circuitry is not shown.

For acquiring multi-parametric signals in-vivo at the fingertips, the experimental setup shown in Figure 3 was used. The OFE and AUX blocks are integrated within a finger clip, while the CF sensor is located on the lower internal surface of the finger clip. A cylindrical force concentrator ensures the pressure is evenly distributed in the CF sensor. Electrical signals from the sensor are transmitted via wires to a plastic casing that contains the rest of the circuitry. The housing is attached to the finger clip to improve its physical durability and facilitate electrical connections between PCBs. The OFE is protected with silicone glue and transparent adhesive tape for easy cleaning and sterilization.

### 2.2. Sensor System Implementation

**Figure 4:**
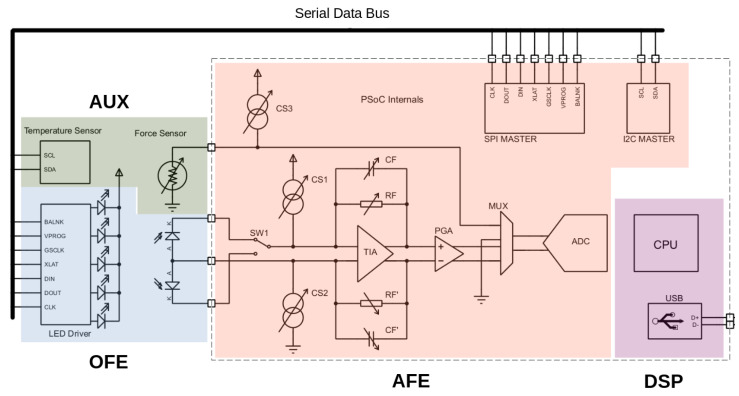
Electrical schematic of the multi-parametric sensor system. Within the dashed lines are the hardware solutions implemented inside the PSoC. The functional blocks are highlighted in different colors.

Figure 4 illustrates the electrical diagram of the system. The PSoC-based circuitry, enclosed by dashed lines, was designed to capture MW-PPG signals accurately while reducing the impact of common mode noise. To accomplish this, all MW-PPG signals are acquired differentially [38]. The PDs measure the back-scattered light intensity and then convert it into a voltage using a Trans-Impedance Amplifier (TIA). A programmable feedback resistor and capacitor select the conversion gain and bandwidth. The selection of PDs is made via the internal analog switch SW1, which allows for the choice of both PDs simultaneously if needed. The TIA output signal is amplified between 0 and +34 dB through a Programmable Gain Amplifier (PGA). The output of the PGA is connected to the Analog–Digital Converter (ADC) via the analog multiplexer MUX. The Delta Sigma ADC is programmed for low-noise, low-speed, and high-resolution (16 to 20-bit) measurements.

The AFE can adjust the gain for each wavelength independently. This is a valuable feature that enhances the signal quality. By changing the gain for each wavelength, the system can compensate for the inherent sensitivity differences between different PDs, resulting in higher-quality signals. To expand the dynamic range of the ADC, a DDCC circuit with two programmable current sources (CS1 and CS2) is used to extract the ambient light and DC of the MW-PPG signal from the TIA input. The DDCC solution can provide a current range of 0–32 µA with a resolution of 0.125 µA. In addition, the CF sensor is driven by a programmable current source (CS3), and the corresponding CF value is calculated in firmware by measuring the voltage drop across the CF via the ADC. Skin temperature is directly obtained from the temperature sensor using a master I2C communication module. The acquired multi-parametric signals are pre-processed by the CPU and transmitted through the internal USB-UART module.

**Figure 5:**
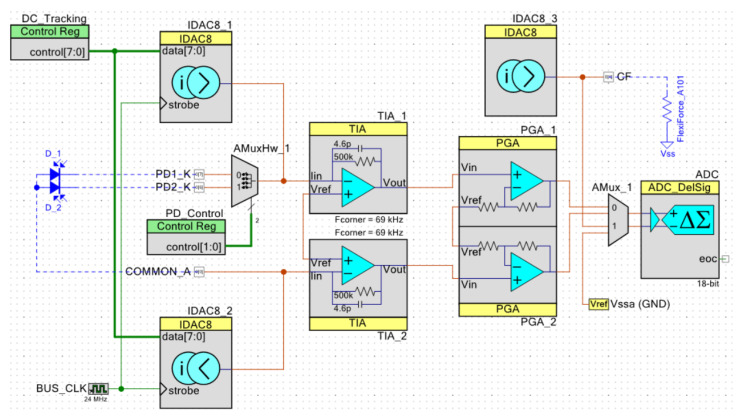
Internal details of the PSoC circuit: AFE section for the conditioning and acquisition of the optical signals and the force sensor.

Figure 5 showcases the internal circuitry of the AFE implemented within the PSoC device. As mentioned, the MW-PPG signals undergo conditioning using a differential configuration. This approach enhances noise immunity and simplifies signal processing, albeit at the expense of increased utilization of the PSoC internal resources. Additionally, in the experimental setup, the fully differential configuration helps mitigate the impact of stray inductance in the photodiode wiring.

TIA_1 and TIA_2 constitute the programmable differential TIA. In turn, PGA_1 and PGA_2 form the differential PGA, isolating the TIA from the ADC. The TIAs and PGAs in the circuit are based on configurable Switched Capacitor/Continuous Time (CS/CT) blocks. Each CS/CT block incorporates a high-bandwidth rail-to-rail operational amplifier. The AFE uses all four CS/CT blocks available in the PSoC Cy8C58xx family. On the other hand, the programmable current sources used for the DDCC circuit are identified as IDAC8_1 and IDAC8_1. At the same time, IDAC8_3 is used to drive the CF sensor. The ADC was configured for an input range of ±2.048 V using the internal reference voltage of 1.024 V ± 0.1%, and the internal buffer is configured as rail-to-rail with a gain of 1.

## 3. Evaluation of the Sensor System

This section presents the evaluation process and the preliminary results obtained. The accuracy of the system in acquiring MW-PPG signals and the performance of the DDCC circuit were evaluated using a proof-of-concept experimental setup. An AECG100 reference waveform generator from WhaleTeq Co., Ltd. (Taipei City, Taiwan) was utilized to assess the acquisition accuracy of the sensor system. The generator was set to produce synthesized PPG signals with wavelengths of 525, 630, and 940 nm, with a pulse period of 1000 ms. The 525 nm reference signal was obtained using the PPG-1R-525 evaluation module connected to the AECG100, while the 630 and 940 nm wavelengths were obtained using the PPG-2R-940 evaluation module. In addition, the electrical output from the testing modules was also recorded using a DS1074Z-S Plus digital oscilloscope from RIGOL Technologies EU GmbH (Gilching, Germany).

The experimental setup designed for this purpose performed in vivo measurements of multi-parametric signals at the fingertip. A healthy 35-year-old male volunteer participated in the study. The volunteer was instructed to remain seated, relaxed, and breathe normally throughout the experiment. The index finger of the left hand was used for all tests, while the arm rested comfortably on a table at heart level. It is worth noting that, based on the Fitzpatrick scale, the skin tone of the volunteer was classified as type V.

### 3.1. Evaluation of MW-PPG Signals Acquisition

**Figure 6:**
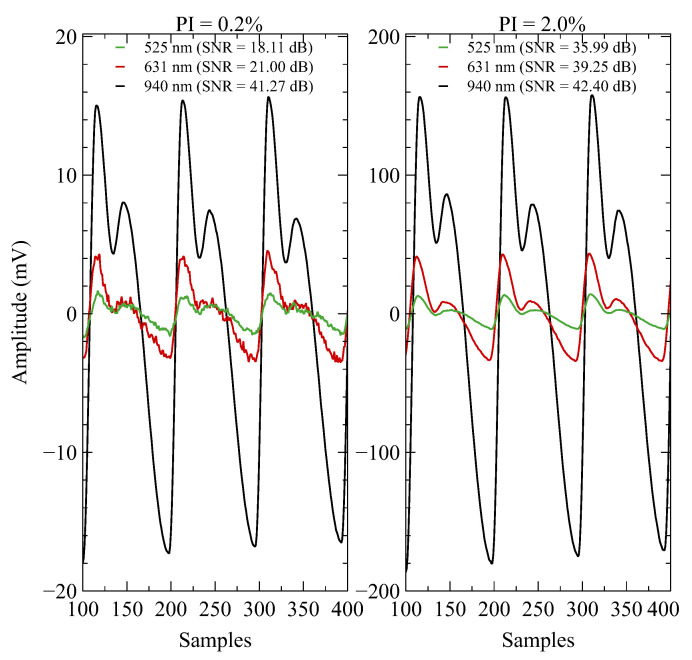
Evaluation of the multi-parametric sensor system to capture reference signals that exhibit different levels of PI. The reference signal with the minimum PI (0.2%) is displayed on the left side, while the reference signal with the maximum PI (2.0%) is shown on the right side. The corresponding SNR values for each wavelength are in the upper part.

The perfusion index (PI) is the gold standard for assessing PPG signal quality [39]. It measures the ratio of pulsatile blood flow to non-pulsatile or static blood in peripheral tissue. It is influenced by various factors such as temperature, CF, skin type, motion artifacts, ambient light, fitness levels, and body fat content [40]. To evaluate the signal acquisition at different PI levels, reference PPG signals with minimum (0.2%) and maximum (2.0%) PI were generated. To ensure accurate signal acquisition, the device under test was placed on evaluation modules. Measures were taken to minimize the impact of ambient light on the generated PPG signal, including obtaining a matte cover to block external light and avoid interference. Furthermore, the device was tested in a controlled lighting environment. During each recording session, the LEDs remained off, and the reference optical PPG signals were recorded one at a time using the PD1. A gain of 250 KΩ was set for the TIA with a compensation capacitor of 4.6 pF, and the PGA gain was set to 2. Signals were acquired with a 100 sps sampling rate and 18-bit resolution. The results indicate correct signal acquisition for both PI levels, as shown in Figure 6. As can be seen, the signal-to-noise ratio (SNR) was lower for a lower PI and shorter wavelengths due to the spectral sensitivity of PD, which is more significant for longer wavelengths. However, the acquired raw signal quality was sufficient to visually identify the main features of PPG signal morphology in both scenarios.

**Figure 7:**
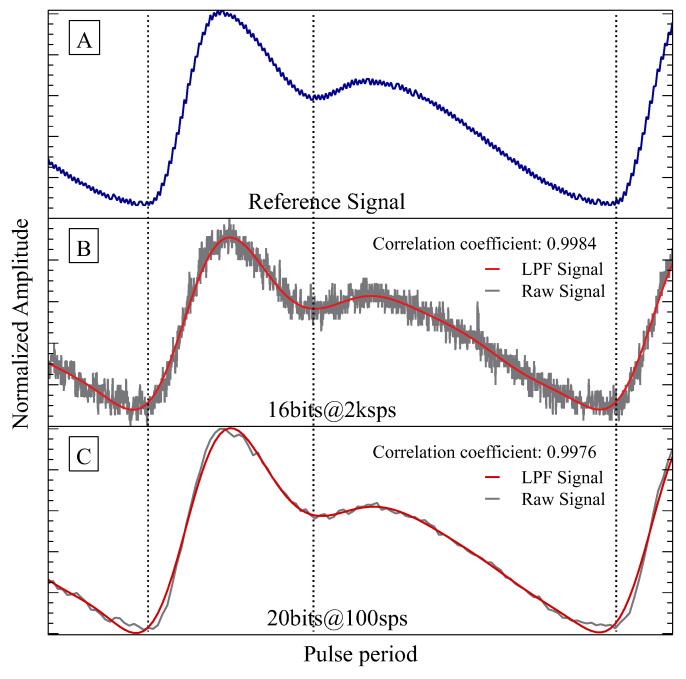
Evaluation of the AFE performance at 525 nm wavelength. (**A**) Reference signal; (**B**) Acquired signal (grey) with 16-bit resolution and 2 ksps sampling frequency; and (**C**) Acquired signal (grey) with 20-bit resolution and 100 sps sampling frequency. The red line represents the low-pass filtered signal for (**A**,**B**). The Pearson correlation coefficients are also shown.

The system performance in capturing PPG signals at different resolutions and sampling frequencies was also evaluated. For this, an optical signal of 525 nm was used as the reference signal (see Figure 7A). In the first test, the sensor system was set to acquire the reference signal with a resolution of 16-bit and a sampling rate of 2 ksps, as illustrated in Figure 7B. The system was then reconfigured in the second test to a resolution of 20-bit and a lower sampling rate of 100 sps, as shown in Figure 7C. To better assess the feasibility of reconstructing the PPG signal under various combinations of sample rate and ADC resolutions, filtered signals were obtained in Python. A fourth-order Butterworth low-pass filter with a cutoff frequency of 10 Hz was applied to the acquired raw signals. The filtered signals are depicted as the red waveform in Figure 7B,C. The Pearson correlation coefficients between the reference PPG signal and the acquired signals were computed and presented in the exact figure. Normalizing and then interpolating the acquired signals ensured that all signals had the same scale and number of data points as the reference signal, making it easier to calculate the correlation coefficients accurately. The correlation coefficients were 0.9984 and 0.9976 for the signals received at ADC resolutions of 16 and 20-bit, respectively.

To optimize the resolution and sampling rate of the ADC for a specific application, it is essential to consider the number of optical channels and the physiological characteristics to be extracted from the PPG signal. Different resolutions and sample rates should be evaluated independently to determine the optimal settings for the system. During our evaluation, the AFE did not induce noticeable disturbances or distortions, except for the quantization noise generated by the ADC. A higher-resolution ADC can capture more signal detail and provide a more accurate representation of the PPG signal. At the same time, a lower sample rate can improve signal stability by reducing noise. However, finding the optimal balance between resolution and sample rate is critical to avoid aliasing or distortion. In our study, the accuracy of the proposed measurement system was confirmed, as demonstrated by a high correlation coefficient (>0.99) between the reference PPG signal and the measured signal. The original morphology of the signal was preserved, as evidenced by the high correlation coefficient obtained. Therefore, the proposed system provides a high-quality raw signal for further analysis using advanced signal processing and signal quality assessment techniques.

### 3.2. Evaluation of the DDCC Circuit

**Figure 8:**
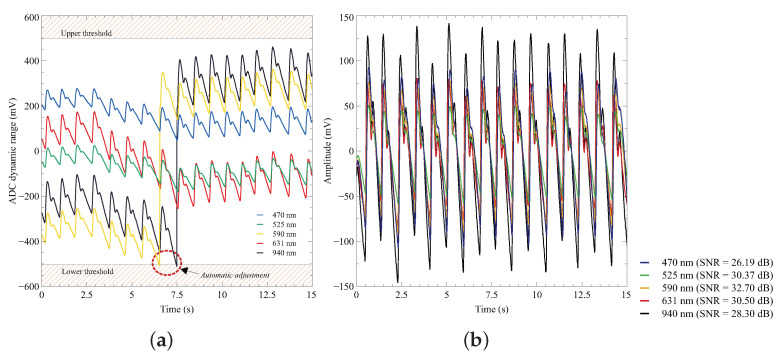
(**a**) Details of the operation of the DDCC circuit while a MW-PPG signal is acquired in real time. The circuit is designed to keep the signals at a pre-configured dynamic voltage range. The point at which the correction circuit is activated to return the 940 nm PPG signals to the predetermined level is signaled. (**b**) MW-PPG signal after being reconstructed and high-pass filtered (Fc = 0.2 Hz).

The successful implementation of a DDCC circuit is vital to achieving high accuracy and SNR in multiplexed MW-PPG schemes. This technique removes most of the DC of the signal. It increases the dynamic range of the ADC, allowing the amplified PPG AC signal to be recorded without analog filtering. This complex process often requires sample and hold circuitry to isolate the signals [41]. The DDCC solution implemented in the proposed system uses a digital-feedback loop to subtract the DC of each wavelength separately. Figure 8a demonstrates the efficiency of the implemented circuit by showing the results of the DDCC circuit action on the MW-PPG signal acquired from the index finger of the volunteer subject. The upper and lower thresholds were set in that range for circuit evaluation and can be adjusted independently according to the needs of the experiment. After adjustment, the signals can be reconstructed using sample-to-sample correction coefficients stored and transmitted along with the acquired data, as demonstrated in Figure 8b.

One crucial point is that appropriate gain settings should be selected to avoid excessive gain in the TIA. This can amplify the current steps injected or extracted by the correction circuit, potentially leading to the saturation of the TIA. Compared to analog filtering, this method avoids introducing non-linear phase distortions into the PPG signal and reduces the number of electronic components.

### 3.3. Evaluation of the CF Measurements

**Figure 9:**
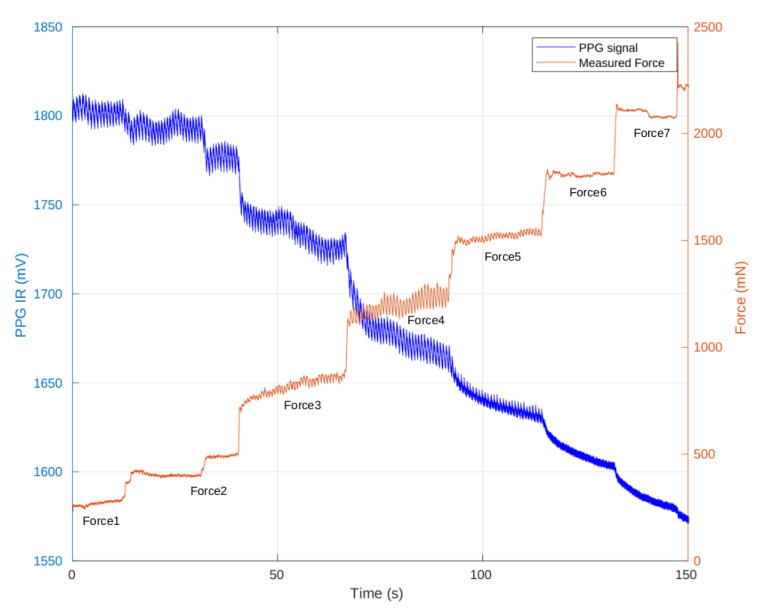
Evolution of the 940 nm PPG signal (blue waveform) compared to CF variations (orange waveform) across seven incremental CF levels.

The CF sensor was calibrated for a CF range of 0–2 N based on literature values [25,42,43]. The performance of the CF sensor was evaluated through an in vivo test. During the test, the force applied to the index finger was incrementally increased in discrete steps until the AC component of the 940 nm wavelength vanished. Figure 9 presents the preliminary results, illustrating the progressive changes in the PPG signal at a 940 nm wavelength and the corresponding force sensor signal across seven incremental levels of CF. The figure showcases the relationship between force levels, PPG signal characteristics (blue waveform), and CF sensor readings (orange waveform). The results indicate that the highest AC amplitude of the 940 nm wavelength was achieved at the CF Force4 level, followed by a gradual decrease in AC amplitude.

**Figure 10:**
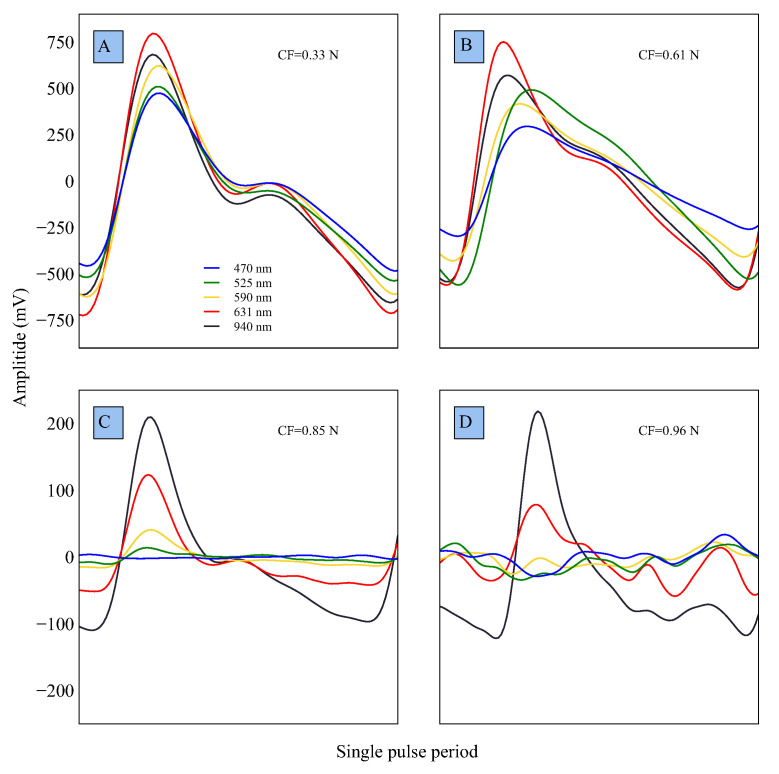
MW-PPG AC components signal contour and amplitude for CF levels of (**A**) 0.33, (**B**) 0.61, (**C**) 0.85, and (**D**) 0.96 N demonstrate the effect of CF on the signal.

Another experiment investigated the impact of four incremental force levels (0.33, 0.61, 0.85, and 0.96 N) on the MW-PPG AC component. The results (Figure 10) show that the distortion of all wavelengths increases with higher applied CF levels compared to the reference (Figure 10A). The effect is more pronounced for shorter wavelengths (470 nm and 525 nm) than longer ones (631 nm and 940 nm), with 590 nm being intermediate.

The CF applied during PPG measurements is crucial in obtaining accurate and reliable signals. Studies have shown that the CF exerted on the skin can significantly affect the PPG signal characteristics, particularly the amplitude of the AC component and the DC level. Our findings are consistent with previous reports in the literature, wherein the optimal level of PPG signal amplitude is typically achieved for medium force values where the transmural pressure is close to zero [25]. This suggests that excessive CF can lead to undesirable signal alterations. The effect of CF on the AC component of the PPG signal is non-linear, with a threshold behavior. Small forces may have little impact on the signal, while larger forces can rapidly distort it. This behavior highlights the importance of carefully controlling the CF to maintain optimal signal quality.

The decrease in the AC signal amplitude with increasing CF can be attributed to the blood flow occlusion caused by vessel compression. This occlusion effect disrupts the pulsatile changes in blood volume, resulting in a reduced AC component. Moreover, the CF also affects the DC level of the PPG signal. A higher CF can alter the DC level, suggesting possible changes in blood perfusion. This further highlights the impact of CF on the overall PPG signal characteristics and the importance of considering the viscoelastic characteristics of the tissue.

Furthermore, the SNR plays a crucial role in PPG measurements. It is important to note that inadequate CF levels can decrease the SNR, adversely affecting the quality and reliability of the acquired PPG signal. This can make it difficult to accurately detect and extract features of interest from the signal, compromising the accuracy of physiological measurements. It should be emphasized that CF effects are most pronounced in the superficial vascular layers. Therefore, special attention must be paid to CF when using shorter wavelengths. The influence of the CF on the superficial layers may be more significant due to the increased susceptibility of blood vessels to compression and possible distortion of the PPG signal. To obtain optimal and reliable MW-PPG measurements, finding a balance in the CF is necessary, avoiding excessive compression of the blood vessels and a significant decrease in SNR.

**Figure 11:**
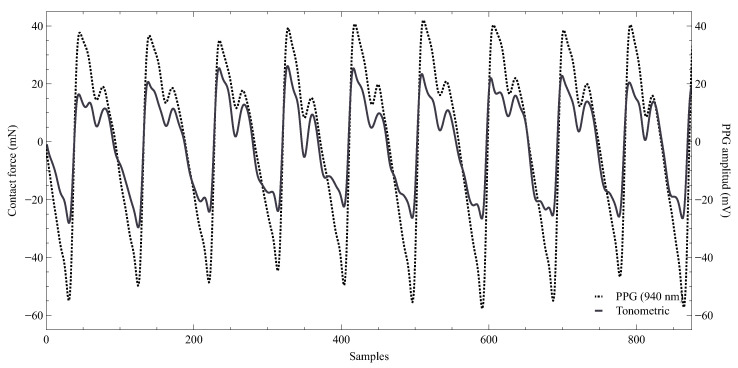
Waveform of the tonometric signal represented by the solid line. For comparison purposes, the AC component of the 940 nm PPG signal is also shown (dotted lines).

The CF sensor output (Figure 11) also provided the tonometric signal, which exhibits similar morphologic characteristics to the pulse waves observed in the PPG signal due to the connection between arterial distension, volume, and arterial pressure. Tonometry is a non-invasive technique to assess pressure in closed vessels throughout the body continuously. For instance, arterial tonometry measures the arteries pressure transmitted through the surrounding tissue, allowing for continuous non-invasive monitoring of the arterial waveform. However, the accuracy of this technology and its integration into standard practice are not yet widely documented [44].

The study results highlight that the FlexiForce A101 force sensor demonstrates adequate sensitivity for contact force measurement and in detecting the tonometric signal. The latter can be used to monitor the propagation of blood mechanical waves following cardiac contraction. Notably, the amplitude and shape of the tonometric signal are contingent on both the force exerted by the sensor on the artery and the viscoelastic properties of the tissue. Nevertheless, continuous sensor sensitivity and accuracy assessment are essential due to possible changes. Additionally, a meticulous evaluation of the mechanical design characteristics is crucial, given their significant impact on the static and dynamic behavior of the CF sensor.

Although there are persistent challenges regarding precision, sensitivity, and sensor placement, developing tonometric devices in conjunction with MW-PG shows potential for improving long-term and continuous blood pressure monitoring. Furthermore, it may enhance other techniques, such as evaluating vascular endothelial function. For future work, a more sensitive sensor and an improved mechanical design would facilitate the successful integration of force sensors into devices. These enhancements would likely improve signal quality, benefiting their application in various health monitoring systems.

### 3.4. Evaluation of the Temperature Measurements

Temperature measurement accuracy is crucial for analyzing MW-PPG signal waveforms and directly impacts peripheral blood vessel perfusion. The resolution and sampling frequency of temperature measurements depends on the sensor type, the thermal time constant, and the specific characteristics to be extracted. Higher frequencies and resolutions are required for evaluating tissue thermal oscillations, while lower sampling rates and resolutions are suitable for determining body temperature. Vascularization at the measurement point also affects temperature measurement results.

**Figure 12:**
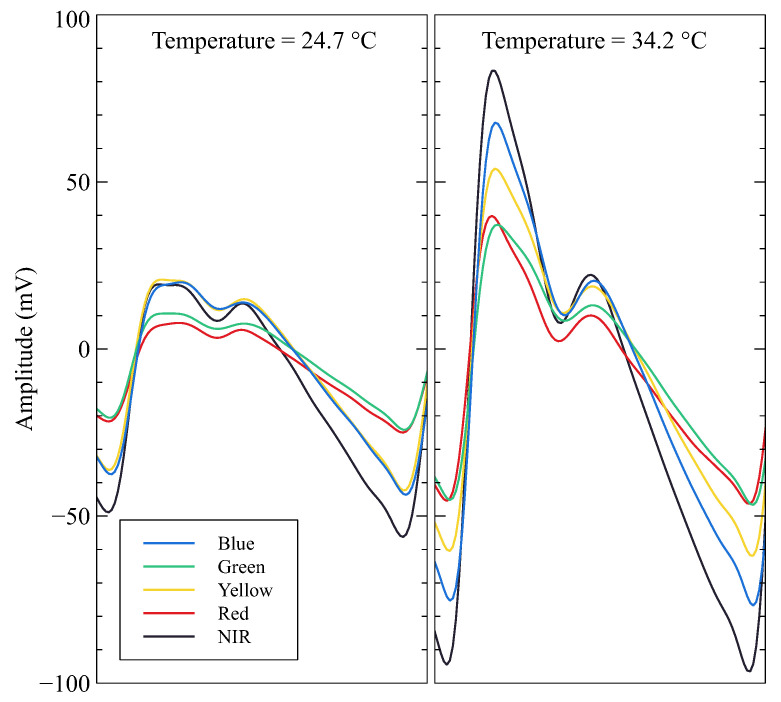
MW-PPG AC components signal contour and amplitude differences comparison for temperature levels of 24.7 and 34.2 °C.

To evaluate the temperature measurements, the volunteer was instructed to immerse their left hand in ice water (0 to 5 °C) for no more than 5 min. After that, the hand was gently dried without applying excessive pressure. Then, the probe was attached to the index finger, and the signals were recorded while the finger naturally heated until the skin temperature stabilized. Figure 12 illustrates how skin surface temperature can significantly influence the amplitude and contour of the MW-PPG signal. A study with a primary sampling frequency of 1 sps and a resolution of 0.0039 °C showed that cooler skin surface temperatures could alter the MW-PPG contour and amplitude, as found by other authors [11,16].

The literature reports wide variations in waveform characteristics with slight changes in temperature at the fingertip, making extracting accurate PAT or PTT values challenging when calculating parameters such as blood pressure from PPG [11]. In contrast, research conducted by Khan et al. revealed that warmer temperatures (32 to 34.6 °C) significantly improved PPG signals quality up to four-fold, enhancing the accuracy of SpO${}_{2}$ estimations [16]. However, the results also indicate that certain signal features, like the dicrotic notch, may be absent, and PPG waveforms may exhibit inconsistency and non-uniformity. For colder temperatures (<20 °C), attempts to enhance LED intensity to maximize absorption and bolster the transmit beam power yielded inconsistent results.

Integrating a high-precision temperature sensor and an effective sampling technique into the designed multi-parametric device allows for accurate temperature measurements. This facilitates a more precise evaluation of how temperature influences MW-PPG signal waveforms and peripheral blood vessel perfusion. This underlines the critical role of accurate temperature measurement in analyzing MW-PPG waveforms and draws attention to the necessity of considering the temperature effect on the signal.

### 3.5. Evaluation of the Multi-Parametric Measurements

**Figure 13:**
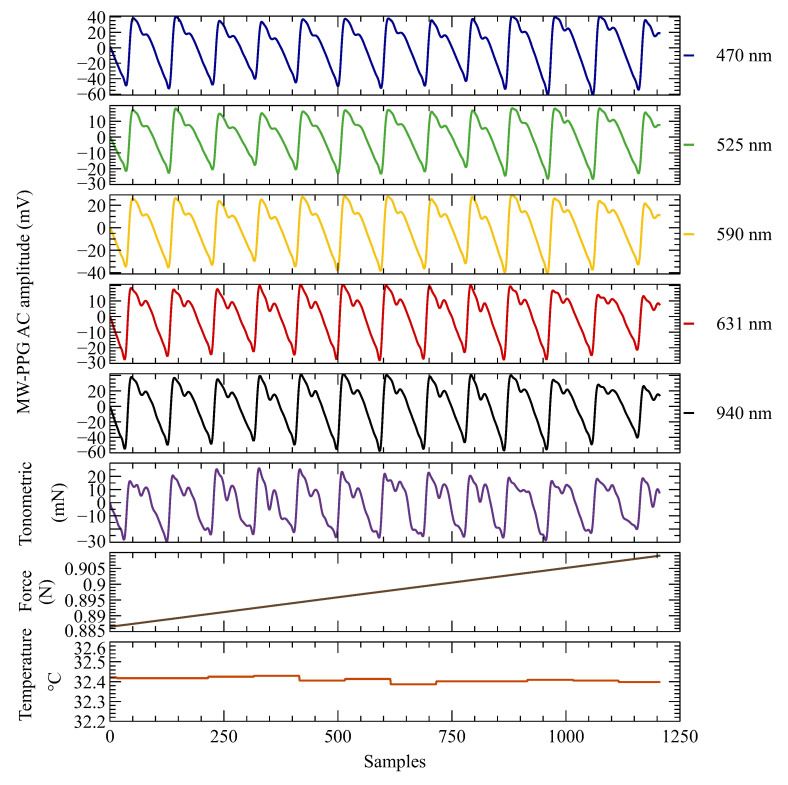
The performance of the designed system was evaluated through in vivo measurements in real time, wherein multiple parameters were recorded simultaneously. The figure shows the AC component of the MW-PPG signal at wavelengths of 470 nm, 525 nm, 590 nm, 631 nm, and 940 nm, as well as the temperature readings, absolute CF, and tonometric signal.

To evaluate the overall performance of the designed system, real-time and synchronized in vivo measurements of the MW-PPG, temperature, and CF signals were performed. At the same time, the contact force was manually and continuously increased. Figure 13 shows a capture of the resulting multi-parametric measurements of the AC component of PPG signals of 470, 525, 590, 631, and 940 nm, temperature, absolute CF, and tonometric signal. MW-PPG signals were acquired at 18-bit ADC resolution, and all LEDs were driven at 20 mA current to maintain the maximum wavelength specified by the manufacturer. The AFE gain was constant for all wavelengths, and all signals were sampled at a center frequency of 100 sps, except for the temperature sensor, which was sampled at 1 sps, synchronized with the primary sampling frequency.

Within the range of CF and temperature shown in the figure, the PPG contour of all wavelengths with pulsatile components present can be visualized, demonstrating the high quality of the signal. The shorter wavelengths, such as 470 and 525 nm, showed an amplitude comparable to the longer wavelengths. However, as a general rule, PDs have inherently lower sensitivity at shorter wavelengths, which tends to skew results compared to other wavelengths with better sensitivity. Further studies are needed to understand this phenomenon better and to determine whether it is related to specific optical properties of the tissue. The tonometric signal is also shown, but as previously discussed in Section 3.3, the sensitivity of the CF sensor may have influenced the morphological variation and signal quality between pulses.

The proposed system offers unprocessed raw data, crucial for research purposes, as previous studies have emphasized [45]. Raw data are essential for testing new signal processing approaches, as many recordings available in databases and advertised as raw signals have in fact been pre-processed and filtered, resulting in the loss of valuable secondary information. Our system provides raw data from MW-PPG and other relevant auxiliary sensors, such as temperature and CF, allowing for a more comprehensive dataset and potentially new insights into measured behaviors. Even in clinical settings, raw signals are usually not accessible, making it challenging to generate a universal dataset. Providing raw data from various sensors, including temperature, CF, and the MW-PPG signal, would be beneficial for testing novel approaches.

## 4. Future Work

The promising results obtained from the proposed multi-parametric system underscore the potential for continued development and refinement in the future. Critical avenues for exploration include integrating a broader range of auxiliary signals to facilitate a more comprehensive investigation into various physical and physiological variables impacting MW-PPG measurements.

A deeper understanding of the factors influencing signal accuracy and reliability is imperative, specifically concerning disturbances of various AC waveform characteristics and their subsequent impact on signal quality indices (SQI). This would allow for the implementation of strategic solutions to minimize the effect of these variables, thus improving the robustness of the measurements and the general reliability of the multi-parametric system, maintaining the integrity of the MW-PPG waveform against external influences. In addition to this, an exploration of wearable-compatible MW-PPG systems will be initiated for non-invasive, continuous monitoring of physiological signals. Key considerations in this regard will be the energy efficiency and practicality of such systems to facilitate their usage in everyday life and clinical settings. As part of these considerations, system size, comfort, and user-friendliness will also be considered to ensure ease of use and user compliance.

Moreover, advanced in vivo experiments will be conducted to better understand how these factors affect the MW-PPG signal in actual physiological conditions. This will not only help improve the accuracy and reliability of the measurements but also contribute to the overall optimization of the multi-parametric system. Finally, in line with advancements in artificial intelligence and machine learning, future work may consider incorporating these techniques for improved signal processing and analysis. Such integration could enable more precise and personalized healthcare monitoring systems, benefiting individual users and the broader healthcare community.

## 5. Conclusions

The presented work represents a significant contribution to non-invasive monitoring techniques by designing and testing a novel multi-parametric sensor system. This system is designed to evaluate the influence of external factors such as CF and temperature in the contour of MW-PPG signals, as well as to provide complete and high-quality raw data. This allows for a detailed examination of the PPG signal contour in different vascular layers to foster new approaches in signal processing. Based on a PSoC device, the system foundations allow for real-time acquisition, reconfigurability, flexibility, scalability, easy integration with small electronics, and high measurement accuracy. Integrating five wavelengths and CF and temperature sensors presents a holistic approach. The tests involved a proof-of-concept device and another integrated into a finger clip for in vivo measurements.

Synthetic PPG signal generation was effectively employed for system evaluation in accurate signal acquisition at various PI levels. In addition, the ability of the system to capture PPG signals at different resolutions and sampling frequencies was evaluated, showing a high correlation with the reference signal. By incorporating a DDCC circuit, the system effectively removed most DC components from the signal, improving the dynamic range of the ADC and reducing non-linear phase distortion. The preliminary in vivo study highlighted competent system design solutions for measuring FC and temperature. It confirmed the impact of these external factor variations on the MW-PPG signal contour.

However, there could be room for improvement. Despite the merits of the system, further validation and testing with larger cohorts under different conditions may be necessary to ensure the robustness of the system. Implementing more advanced signal processing algorithms to handle the acquired data could also be considered. Overall, this work is critical for future advances in PPG research, furthering the development of accurate, reliable, and standardized systems for clinical practice and non-invasive vascular physiology monitoring. Understanding various factors that affect the PPG signal is crucial to the progress of the research. This system forms a solid foundation for designing metrological evaluation methodologies for PPG-based handheld devices. It also helps translate research into practical applications that align with regulatory requirements for medical device certification.

## Data Availability

Data sharing not applicable.
